# Shedding Light on Colorectal Cancer: An In Vivo Raman Spectroscopy Approach Combined with Deep Learning Analysis

**DOI:** 10.3390/ijms242316582

**Published:** 2023-11-21

**Authors:** Maria Anthi Kouri, Maria Karnachoriti, Ellas Spyratou, Spyros Orfanoudakis, Dimitris Kalatzis, Athanassios G. Kontos, Ioannis Seimenis, Efstathios P. Efstathopoulos, Alexandra Tsaroucha, Maria Lambropoulou

**Affiliations:** 1 2nd Department of Radiology, Medical School, National and Kapodistrian University of Athens, 11527 Athens, Greece; mariakouri90@gmail.com (M.A.K.); spyratouellas@gmail.com (E.S.); dimitriskalatzis@gmail.com (D.K.); stathise@med.uoa.gr (E.P.E.); 2 Medical Physics Program, Department of Physics and Applied Physics, Kennedy College of Sciences, University of Massachusetts Lowell, 265 Riverside St., Lowell, MA 01854, USA; 3 Physics Department, School of Applied Mathematical and Physical Sciences, National Technical University of Athens, Iroon Politechniou 9, 15780 Athens, Greece; mariakarnach@gmail.com (M.K.); s.orfanoudakis@inn.demokritos.gr (S.O.);; 4 Medical School, National and Kapodistrian University of Athens, 75 Mikras Assias Str., 11527 Athens, Greece; iseimen@med.uoa.gr; 5 Laboratory of Bioethics, School of Medicine, Democritus University of Thrace, 68100 Alexandroupolis, Greece; 6 Laboratory of Histology-Embryology, School of Medicine, Democritus University of Thrace, 68100 Alexandroupolis, Greece; mlambro@med.duth.gr

**Keywords:** SCID mice model, colorectal cancer, Raman spectroscopy, portable Raman probe, transfer learning analysis, tissue classification

## Abstract

Raman spectroscopy has emerged as a powerful tool in medical, biochemical, and biological research with high specificity, sensitivity, and spatial and temporal resolution. Recent advanced Raman systems, such as portable Raman systems and fiber-optic probes, provide the potential for accurate in vivo discrimination between healthy and cancerous tissues. In our study, a portable Raman probe spectrometer was tested in immunosuppressed mice for the in vivo localization of colorectal cancer malignancies from normal tissue margins. The acquired Raman spectra were preprocessed, and principal component analysis (PCA) was performed to facilitate discrimination between malignant and normal tissues and to highlight their biochemical differences using loading plots. A transfer learning model based on a one-dimensional convolutional neural network (1D-CNN) was employed for the Raman spectra data to assess the classification accuracy of Raman spectra in live animals. The 1D-CNN model yielded an 89.9% accuracy and 91.4% precision in tissue classification. Our results contribute to the field of Raman spectroscopy in cancer diagnosis, highlighting its promising role within clinical applications.

## 1. Introduction

Raman spectroscopy is a powerful analytical technique used to gather highly specific information about the short-range molecular vibrations of materials. It involves the excitation of a sample via monochromatic radiation and the detection of the inelastic scattered light. The rich molecular information that exists within the analysis of Raman spectra has led to Raman spectroscopy being viewed as a widely applied and deeply useful tool in the fields of medical, biochemical, and biological research. The ability to detect tissue alterations and the pathological progress of diseases offers significant attention to Raman spectroscopy and eventually classified it as a potential method for non-invasive cancer diagnosis [1].

In the case of colorectal cancer (CRC), various ex vivo studies have been focusing on the capability of Raman spectroscopy to discriminate cancerous from non-cancerous samples while simultaneously providing the molecular information of the colorectal tissue with high specificity and sensitivity [2,3]. The level of accuracy in cancer detection combined with the ability to provide a rapid, label-free diagnosis, as well as the real-time assessment of tissue during surgery, biopsy, or endoscopy procedures, has brought Raman techniques into the spotlight of the clinical field [4]. Furthermore, modern innovations such as portable Raman systems combined with fiber-optic Raman probes have enabled even further research on colon cancer during colonoscopy [5,6] and have offered the ability to identify cancerous and normal tissue margins in vivo [7]. 

The development of such advanced systems is now offering Raman spectrum assessment with high effectiveness and specification on biologically complex living organisms. The effectiveness of miniaturized Raman endoscope systems [8], the simultaneous Fluorescence–Raman Endoscopy techniques [9], fluorescence and white light image-guided Raman spectroscopy approaches for margins detection [10], and Surface Enhanced Resonance Raman Scattering with NanoParticle imaging [11] can be assessed in vivo, with the aid of laboratory animal models. In the field of Raman spectra data analysis, the utilization of transfer learning models, such as that of one-dimensional Convolutional Neural Network (1D-CNN), has shown great promise. Notably, our prior ex vivo Raman study on human colorectal cancer (CRC) samples, as detailed in [12], serves as a testament to the effectiveness of this approach, achieving an accuracy rate of 88.7% in the crucial task of distinguishing cancerous from normal tissues.

In this paper, a portable Raman probe spectrometer was tested for in vivo localizing colorectal cancer malignancies from normal tissue margins on immunosuppressed mice. The spectral differences between the normal and cancer specimens were investigated in detail based on the average spectra and their subtraction. Principal component analysis (PCA) was performed on the entire dataset in order to visualize the classification ability between normal and cancer datasets, as well as to highlight their biochemical differences by using loading plots. A transfer learning model based on a one-dimensional convolutional neural network (1D-CNN) was employed for the Raman spectra data analysis with the aim of assessing the classification accuracy of Raman spectra in live animals, thus moving another step closer to the clinical translation of the spectroscopic Raman techniques.

## 2. Results

### 2.1. Histological Evaluation

**Figure 1:**
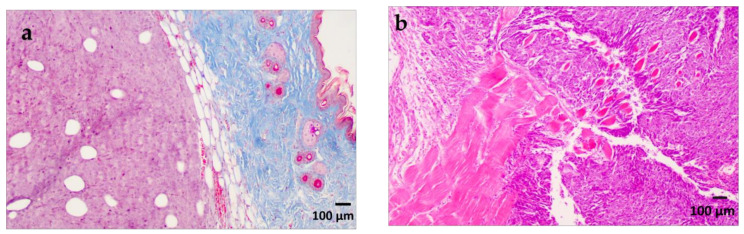
(**a**). Moderate to low differentiated subcutaneous tumor. Masson’s trichrome staining X 100. (**b**). Moderate to low differentiated subcutaneous tumor with muscle invasion. H&E staining X 100.

**Figure 2:**
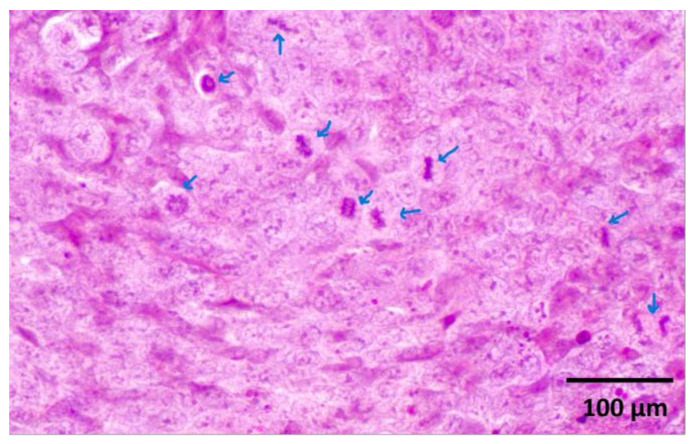
Increased atypical mitotic figures (arrows). H&E staining X400.

**Figure 3:**
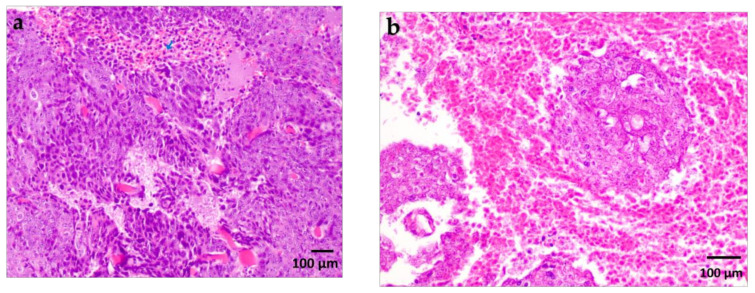
(**a**). Tumoral necrosis with hemorrhage and tumor-infiltrating cells (arrows). H&E staining X 100. (**b**). Intratumoral and peritumoral necrosis and hemorrhage. H&E staining X200.

**Figure 4:**
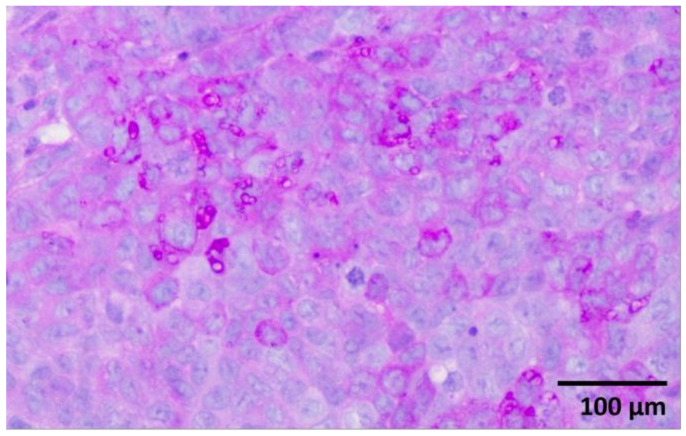
PAS-positive tumor cells (mucin-producing tumor cells with purple-magenta color). PAS staining X 400.

Histological examination revealed a palpable subcutaneous tumor that was a moderate to low differentiated colon carcinoma, depicted in Figure 1a,b; it thus provided indispensable evidence for their classification in the experimental spectroscopic analysis. Each tumor mass was surrounded by fibrous stroma, as indicated by the black arrow in Figure 1a. The tumor cells were enlarged with round or oval nuclei, and the mitotic activity was increased, as illustrated in Figure 2. The central regions of the tumors were necrotic with hemorrhage, tumor-infiltrating cells, and macrophages, as can be observed in Figure 3a,b. Finally, the Foci of PAS-positive mucin-producing tumor cells are also observed and illustrated in Figure 4. 

### 2.2. Raman Spectra Characterization

Generally, a Raman spectrum provides information about the vibrational modes of molecules in biological samples. Therefore, it can reveal the molecular compositions and structures of tissues via their distinct spectroscopic fingerprint. The initial step in spectroscopic analysis involved the careful study of both normal and cancerous spectra, focusing on the identification of Raman modes in each. Subsequently, a comparison of the intensities of the two spectra was performed via subtraction. To ensure statistical precision in achieving these objectives, the average preprocessed spectra were calculated.

**Figure 5:**
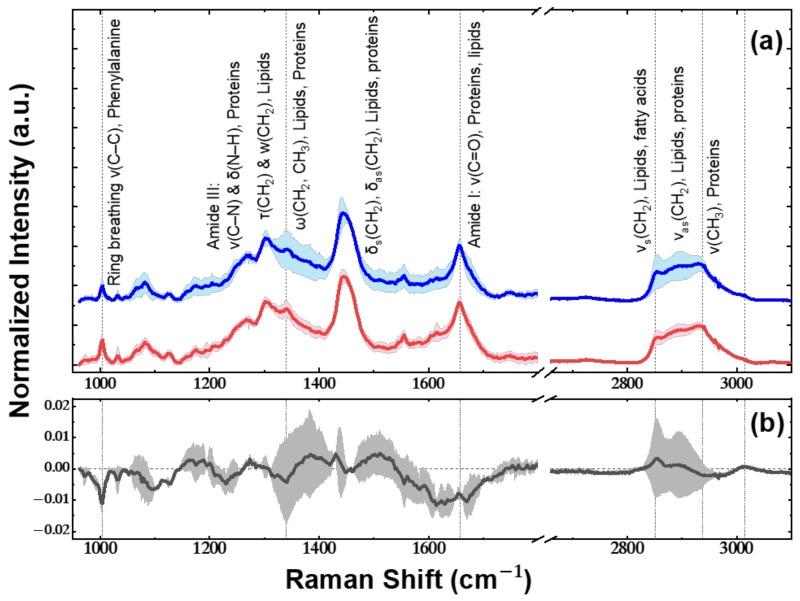
(**a**) Mean normalized spectra of 100 control (blue line) and 140 cancerous (red line) samples. The main vibrational bands and associated molecules are marked above the corresponding peaks. (**b**) The difference spectrum (black line) and vertical dashed lines point to important differences. Standard deviation (±SD) is shown in soft color. The region 1800–2660 cm^−1^ is Raman-silent for biological materials; thus, it is omitted from the spectra presentation.

The average processed spectra for each sample type (control or cancer) are presented in Figure 5a, along with the corresponding standard deviations (±SD). The average of 100 control sample spectra is depicted in blue, while the average of 140 cancer spectra is in red. Raman modes and their attribution are noted in Figure 5a with the identification of the peaks based on our previous ex vivo study, which focused on human CRC [2]. In order to adequately compare the subtle intensity differences between the mean normal and mean cancerous spectra (as depicted in Figure 5a), the difference (normal–cancerous) spectrum was extracted and is displayed in Figure 5b. In Figure 5b, the important differences are pointed with vertical dashed lines and arise from variations in the molecular composition, structure, and density of molecules within the two types of tissues.

**Figure 6:**
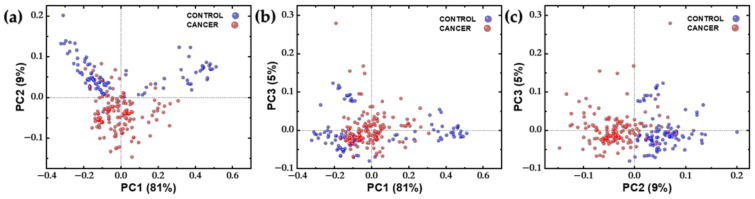
Score plot of the PCA analysis of Raman spectra of control (blue points, n = 100) and cancer samples (red points, n = 140). (**a**) PC1 versus PC2. (**b**) PC1 versus PC3. (**c**) PC2 versus PC3.

**Figure 7:**
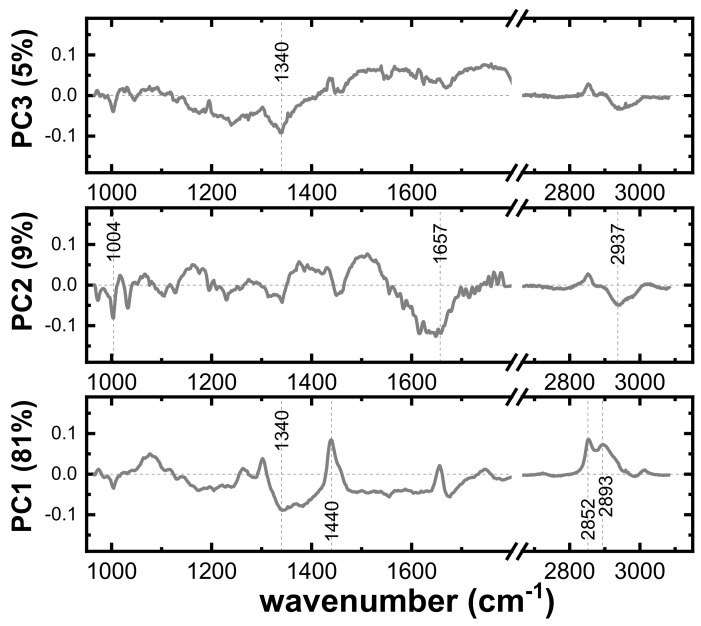
Loading plots of PC1, PC2, and PC3 (weights for PCs versus wavenumber). Maximum and minimum values of weights indicate the wavenumbers, mostly contributing to the differentiation. Vertical dashed lines point to the most intense positive or negative peaks.

Additionally, principal component analysis (PCA) was performed on the spectral dataset in order to visualize the data in a reduced dimensional space and to inspect whether the dataset exhibits good classification potential. Both Score and Loading plots were extracted from this analysis and are depicted in Figure 6 and Figure 7, respectively. For plotting the Score Plots of PCA, the three first PCs were used because they covered the most variance in the datasets (variations: PC1 = 81%, PC2 = 9%, and PC3 = 5%). In these plots, each point represents a single spectrum, and are colored based on the predefined class they belong to (red: cancer; blue: control). The loading plots of PCs in Figure 7 represent the weights of the PCs as functions of the wavenumbers. The highest weights indicate the wavenumbers (or variables), mostly contributing to the differentiation.

### 2.3. Raman Spectra Classification

The results presented in Table 1 demonstrate the performance of the 1D-CNN transfer learning model in classifying normal and cancerous specimens in the mice model. Notably, the model exhibits a high degree of accuracy at the level of 89.9%. Moreover, the substantial recall rates of 85% for the control group and 92.8% for the cancer group emphasize the model’s capacity to identify most of the relevant instances in each class, which is vital for medical diagnosis and cancer localization.

**Figure 8:**
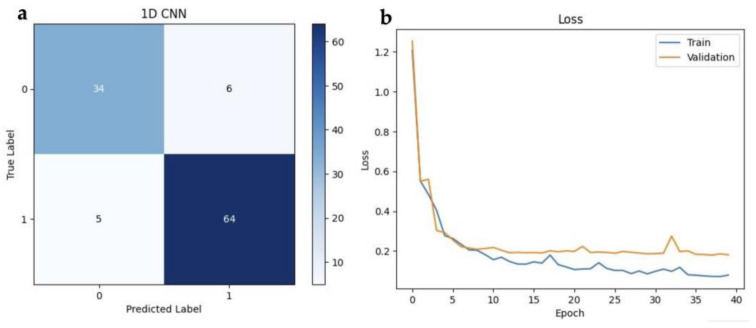
(**a**) Confusion matrix of 1D-CNN. The labels 0 and 1 correspond to normal and cancerous, respectively. (**b**) Training and validation loss of 1D-CNN.

A confusion matrix (Figure 8a) provides a detailed breakdown of the model’s predictions and their correspondence with the actual outcomes. This matrix consists of four key components: true positives (correctly predicted positive instances), true negatives (correctly predicted negative instances), false positives (incorrectly predicted positive instances), and false negatives (incorrectly predicted negative instances).

In the plot (Figure 8b) of validation loss over epochs, the performance of the model was observed during training. The *x*-axis represents the number of training epochs, while the *y*-axis represents the validation loss, which quantifies how well the model generalizes unseen data. As training progresses, the loss steadily decreases, reflecting the model’s improvement in capturing underlying patterns in the data. The final validation loss at Epoch 40 demonstrates the model’s ability to make accurate predictions on new, unseen data, which is a crucial measure of its generalization capability.

## 3. Discussion

Our study relies on the analysis of the minor spectroscopic differences between normal and cancerous CR mice tissues. It was thus imperative to verify independently via histopathological analysis that all the developed tumors constitute cancerous tissues and define their characteristics.

Regarding the spectral analysis, as can be derived from the results (Figure 5a,b), both mean spectra, cancerous and normal, exhibit identical peak positions and display similar spectral profiles. They are rich in peaks assigned to vibrations of tissue compounds such as Raman bands of lipids, proteins, and amino acids. The main bands at 1004, 1256, 1305, 1340, 1445, 1657, 2852, 2884, and 2937 cm^−^^1^ and their assignments noted in Figure 5 are also present in the Raman spectrum of human colorectal tissue studied in our previous work [2]. This correlation to the results of human colon cancer samples is also depicted in the recorded intensity differences, a fact that leads to the confirmation that the mouse model is a valid and representative approach for differentiating normal with cancerous tissue and accurately detecting their margin limits. Based on the difference spectrum (Figure 5b), the Amide I Raman band (Proteins and Lipids) at 1657 cm^−^^1^ is narrower in the case of the normal spectrum, which is a spectral feature characteristic of the adipose tissue spectrum. The expected higher proportion of lipids in normal tissues was also confirmed by the Raman bands at 2852 cm^−^^1^ and 3015 cm^−^^1^ [13], which have a higher normalized intensity than the corresponding spectra in cancer. Furthermore, negative bands at 2937, 1657, 1340, and 1004 cm^−^^1^ indicate that the Raman bands of proteins and amino acids have a higher normalized intensity in the cancer spectrum. Therefore, the expected higher protein content in cancer tissues has also been confirmed. Weak bands in the difference spectrum are also placed at 1094, 1230, 1390, and 1511 cm^−^^1,^ but they do not correspond to particular Raman bands and instead are derived from a marginal difference in the overall shape of the spectral profiles.

Regarding the PCA results, firstly, it is evident from the score plots that the data are fairly well separated, with the most distinct separation observed in the PC2-PC3 plot, followed closely by the PC1-PC2 plot (Figure 6). Moreover, in the three plots of Figure 6a–c, it can be clearly observed that the cancer points are less dispersed, indicating that the homogeneity of the cancerous tissue is reflected in the corresponding spectra. Loading plots of PCs in Figure 7 provide insights into the contribution of different variables (wavelengths) to the principal components. Based on these plots, the PC1 is associated with the variables 1340, 1440, 2852, and 2893 cm^−1^, which are spectroscopically assigned mainly to lipids. This is why a group of points in the PC1–PC2 score plot is separated to the right of the 2D PC space, as all these points correspond to “lipids” spectra. For the loading plot of PC2, negative peaks at the wavenumbers 1004, 1657, and 2937 cm^−1^ have the highest intensity, indicating that the corresponding protein Raman modes have a significant contribution in grouping along the PC2 axis in the score plots (Figure 6a,c). The PC3 loading plot has only one noticeable peak at 1340 cm^−1^. In summary, the best grouping of the score points is along the PC2 axis and consequently the most important Raman bands for the differentiation between cancerous and normal spectra are at 1004 (Ring breathing ν(C–C), Phenylalanine), 1657 (Amide I: ν(C=O), Proteins, lipids), and 2937 cm^−1^ (ν(CH_3_), Proteins), which are corroborated with the frequencies of interest in the difference spectrum (Figure 5b).

The experimentation with the 1D CNN architecture involved the “freeze-then-train” technique, and after several iterations, it was found that the best results were achieved when freezing all layers except the dense layers. This outcome verified that there is a high degree of similarity between the human data, which was used to pre-train the model and mice data in our study. The performance metrics of the 1D-CNN model with transfer learning demonstrate promising results, particularly in the context of cancer localization. With an accuracy rate of 89.9%, the model accurately predicts almost 90% of all cases, showcasing its proficiency in distinguishing cancerous from normal tissue. The precision and recall rates for the cancer group class are 91.4% and 92.8%, respectively, indicating the model’s ability to correctly identify true cancer cases while minimizing false positives. Additionally, the F1 score of 92.1% further validates the model’s effectiveness in achieving a balance between precision and recall. Overall, these results highlight the model’s performance in accurately diagnosing and localizing cancerous tissue, thus constituting a valuable tool in clinical praxis. 

It is worth mentioning that in any experiment involving laboratory animals, inherent limitations are present due to the controlled nature of the environment and the unique characteristics of the animal models. The use of SCID mice, which lack a functional immune system, raises concerns about the ability to fully replicate the intricate interactions between tumors and the immune system within a living organism. The controlled environment of a laboratory and factors like animals’ diet and level of stress pose challenges for accurate experimental replication in future studies. On the other hand, Raman probes combined with portable spectrometers applied in vivo adopt instrumental limitations regarding the spectroscopic and lateral resolution. Furthermore, a standard limitation of RS is the subtle spectroscopic differences between a normal and cancerous state that require the analysis of multiple datasets in order to improve the evaluation metrics.

## 4. Materials and Methods

The implementation of the research procedure includes the pilot application of the biphotonic tool in a preclinical environment and, more precisely, in situ, in laboratory animals. The experimental protocol involved the use of a human cancer cell line, the development of xenografts on SCID (severe combined immunodeficient) mice, the histological examination, and the Raman spectra acquisition and analysis; it is described in detail as follows.

### 4.1. Cell Culture 

The colon cancer line HCT 116 was obtained from ATCC (American Type Culture Collection). Cells were cultured in McCoy’s 5a Medium Modified (ATCC, cat number 30–2007) supplemented with 10% Fatal Bovine serum (FBS) (GIBCO Life Technologies Inc, Gaithersburg, MD) and 1% penicillin/streptomycin. The cultures were maintained in the medium and cultured in a humidified (37 °C, 5% CO_2_) incubator, grown in 75 cm^2^ culture flasks, and passaged when they reached 80% confluence. The cell number was estimated by using hematocytometry after detaching from the flask using 0.25% (*w*/*v*) Trypsin-EDTA solution.

### 4.2. Animal Model

Twenty male, immunocompromised SCID (NOD.CB17Prkdcscid) mice aged approximately 6 weeks old were used. 

The animals were divided into two groups:

1. Group C (control group), where no cancer cells would be injected, only 200 μL of cell culture media, and was composed of 8 animals.
2. Group Ca (cancer group), where tumorigenic potential would be assessed. The group was composed of 12 animals.

All animals were weighed and presented an initial weight range of 25 to 30 g, while the final weight for the animals of the Ca Group ranged from 24 to 29 g.

All experiments were conducted in the licensed facilities of the Laboratory Animal Breeding Institute of the Institute of Biosciences and Applications of the National Center for Natural Sciences Research “Democritos”, Athens (license number (experimentation) EL25BIOexp039).

### 4.3. Subcutaneous Injection of Cancer Cells/Tumor Inoculation

(a) Materials and Reagents
  - Cancer cells of HCT 116 cell line;
  - SCID (NOD.CB17Prkdcscid) mice;
  - Cell reagents (e.g., PBS, Trypan Blue, etc.).

The cell cultured was measured via hematocytometry and resuspended at a concentration of 1.0 × 10^6^/mL before the animal injection.

For inoculation of the cancer cells (HCT116), the dorsal surface inoculation model was selected. The procedure was performed as follows:

- The entity of the mice was anesthetized using a solution of Ketamine: 80–120 mg/kg and Xylazine: 5–10 mg/kg.
- I. Local antisepsis of the skin area was performed.
- The injection of the cell suspension was performed with a 1 cc syringe and a 27G X1/2 needle. Initially, the cells were retracted without the needle. Subcutaneous administration was performed in the dorsal area.
- The injection of the HCT116 cell solution (1.0 × 10^6^/mL) remained slow while the administered volume did not exceed 200 µL per mouse. The injections ended up developing a single bubble of cells under the skin in order to avoid excessive spreading of the administered cells.
- For Group C (control group), 200 μL of plain cell culture media was injected using the same proportions and handling techniques as described above.

### 4.4. Raman Spectra Acquisition

**Figure 9:**
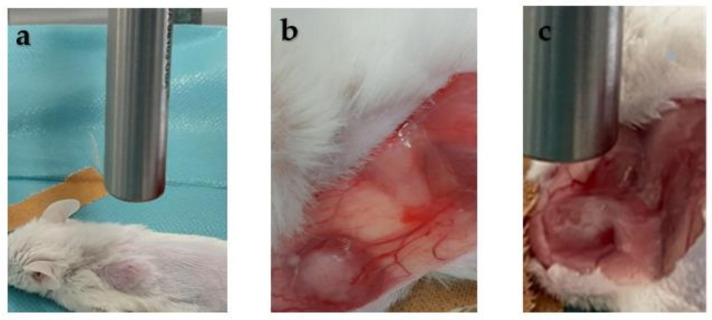
(**a**) Experimental setup. (**b**) Skin incision was performed in the center of the visible neoplastic mass to unveil it. (**c**) Placement of the Raman probe for spectrum acquisition.

When the tumors approached the size of 100 mm^3^, approximately 8–14 days after inoculation, a surgical incision was made at the site of tumor growth. Then, a skin incision was performed at the length of 1.5–2 cm in order to uncover the tumor from the skin layer and thus allow Raman spectra recording using the Raman probe depicted in Figure 9. 

For the Raman measurements, we employed a MarqMetrix All-in-One portable Raman system, which was equipped with a 785 nm solid-state laser. The spectral resolution averaged 6.5 cm^−1^ across a spectral band range from 100 to 3200 cm^−1^. In order to focus the laser beam on the tissues and obtain the Raman signal remotely, a sterilized proximal BallProbe receiver was used. The probe had a sapphire spherical lens for short-range measurements, with the ideal probe–sample distance (optimal signal; lowest luminosity) at 8 mm. The spot beam diameter was 100 μm, and the total length of the probe with the optical fibers was 1.0 m.

**Figure 10:**
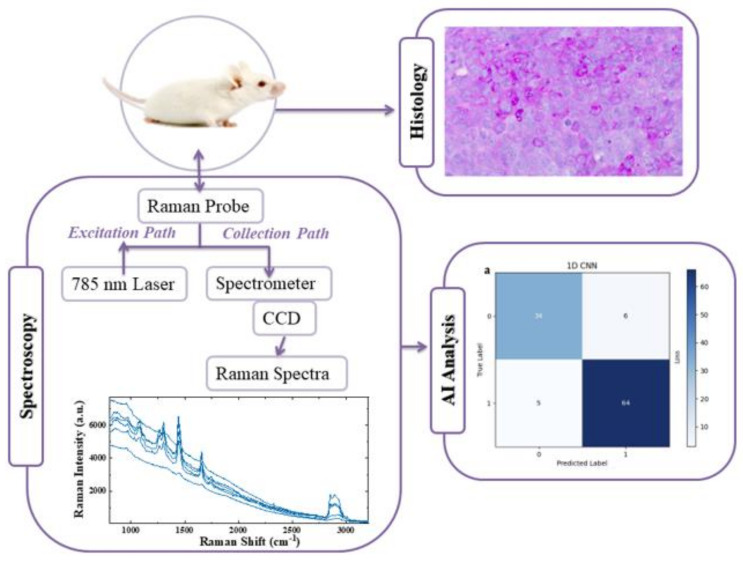
Schematic illustration of the experimental in vivo analysis of the mice models, including histological analysis, Raman spectra acquisition via a Raman probe coupled with a portable Raman spectrometer and AI transfer learning analysis of the data.

The measurements were conducted at a laser power of 300 mW, which was tested to be non-destructive to the animal tissues. The tissues were pre-irradiated for 60 s to reduce the autofluorescence background signal. Then, the Raman signal was averaged over 20 replicate acquisitions of 3.0 s each and was corrected by subtracting the signal recorded without laser irradiation by keeping all the other experimental conditions equivalent. In total, the duration of each spectrum was two minutes. In order to perform statistical analysis, several nearby spots were examined subsequently, with a total measurement time for each animal about 30 min. Finally, the background was removed numerically. Figure 10 shows a schematic illustration of the experimental steps followed for the in vivo Raman analysis in mice.

### 4.5. Histological Examination

After the spectra acquisition, the mice were euthanized, and the tumors were surgically excised in order to perform histological analyses of the samples. 

Specimens were dissected properly and were fixed immediately in 10% formaldehyde, then embedded in paraffin wax, sectioned serially, and stained following standard protocols for hematoxylin and eosin (H&E), Periodic acid–Schiff (PAS) and Masson’s trichrome staining. At the end of the histological procedure and histochemical staining, the slides were mounted and examined under a Nikon Eclipse 50i microscope (Nikon Corporation, Tokyo, Japan).

### 4.6. Spectra Processing and Data Analysis

#### 4.6.1. Spectroscopic Data Analysis

The acquired Raman spectra were preprocessed following three steps: (1) the removal of spectral regions that were dominated by parasitic signals, (2) background subtraction, and (3) normalization. Firstly, all spectra were reduced in size by keeping only the 990–3200 cm^−1^ spectral region that mainly includes tissue-derived Raman signals. The spectral range from 100 to 990 cm^−1^ included Raman bands of the sapphire ball lens of the Raman Probe (417 cm^−1^, 451 cm^−1^, 577 cm^−1^, and 750 cm^−1^) and parasitic interference signals from the detector that confused the spectral analysis. After that, the background signal, dominated by autofluorescence, was corrected using the statistics-sensitive nonlinear iterative peak-clipping (SNIP) algorithm (45 iterations). Finally, the spectra were normalized using vector normalization to allow comparison and improve presentation of the spectra. We utilized the preprocessed spectra both to perform spectroscopic analysis on the mean spectra and to apply principal component analysis (PCA). PCA was applied using the software Orange: Data Mining Toolbox in Python [14]. The wavenumbers from 990 cm^−1^ to 1800 cm^−1^ and from 2660 cm^−1^ to 3100 cm^−1^ were used as variables, and the Score and Loading plots were extracted.

#### 4.6.2. One-Dimensional CNN Transfer Learning

**Figure 11:**
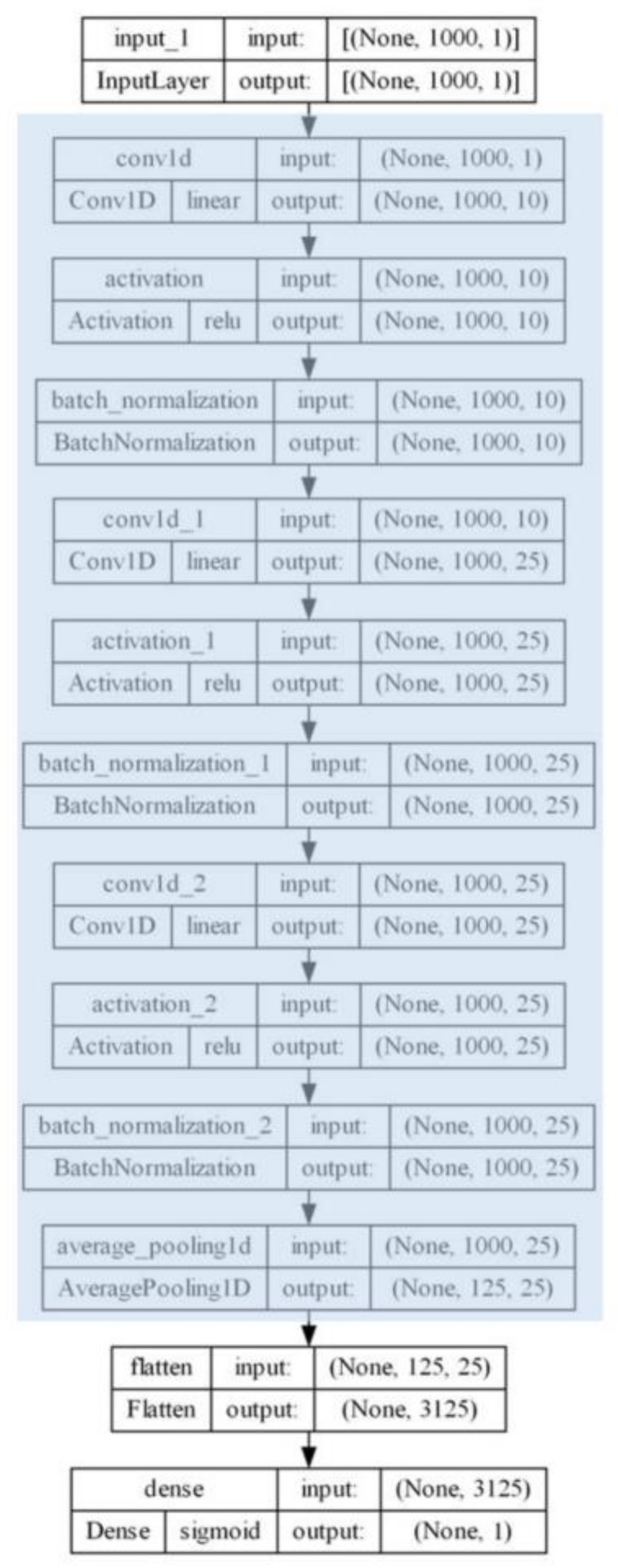
One-dimensional convolutional neural network model architecture. Blue indicates the layers that were frozen for the transfer learning procedure.

In this experiment, the aim was to classify normal and cancerous tissue in Raman spectra of mice samples by employing a 1D-CNN model (Figure 11) with a transfer learning technique to effectively address and mitigate the issue of overfitting. In the pretraining phase, an extended dataset from our previous work was employed [12], composed of 442 Raman spectra of human tissues in total: 221 from normal and 221 from cancerous colorectal tissue samples. Regarding the training strategy, the “freeze then train” technique was used. In this approach, most of the pre-trained model’s layers were kept fixed or “frozen”, and the training was allowed on the last dense layer. In carrying this out, the knowledge and patterns learned by the model from its previous training tasks can be retained. Thereinafter, the last layer was trained to adapt the model’s output to our specific problem. This strategy is valuable because it can save training time and prevent overfitting. In total, 240 spectra were recorded from the mice model (100 normal and 140 cancerous samples). Of the prepossessed data, 60% was used for fine-tuning the model, and the remaining 40% was used for testing and evaluation. Because of the limited dataset size, comprising only 240 samples, 40% of the data was chosen to be allocated for testing. The decision to reserve a relatively larger portion for testing was based on the need to ensure a robust evaluation of our 1D-CNN model’s performance. Additionally, we employed stratified sampling to ensure balanced class representation. Our dataset originally consisted of 100 samples from the normal tissue and 140 samples from the cancerous tissue. Via the application of stratification, we allocated 40 samples from the first class and 69 samples from the second to the test set. This approach maintains a proportional representation of both classes in the testing subset, which is crucial for obtaining accurate and meaningful performance metrics for our 1D-CNN model.

The performance of the 1D-CNN model was assessed using several evaluation metrics, including:

- Accuracy: measures the overall correctness of the model’s predictions and is expressed as the ratio of the correctly predicted samples to the total samples.
- Recall: also known as the true positive rate or sensitivity, quantifies the model’s ability to correctly identify positive instances. It is calculated as the ratio of true positives to the sum of true positives and false negatives.
- Precision: also called positive predictive value, evaluates the model’s accuracy when it predicts a positive instance. It is calculated as the ratio of true positives to the sum of true and false positives.
- F1 Score: the harmonic mean of precision and recall. It provides a balanced measure of the model’s performance, particularly when dealing with imbalanced datasets.

Additionally, the loss and accuracy were visualized during the training process. These training curves will allow to monitor the model’s performance over epochs and provide evidence of whether or not the model is overfitting. By observing these training curves, we can ensure that our model generalizes well to the data and does not memorize it, adding an extra layer of confidence to our evaluation process.

## 5. Conclusions

In conclusion, our research provides valuable insights into the spectroscopic differences between normal and cancerous tissue in laboratory animal models of colorectal cancer. Via a comprehensive analysis of Raman spectra, we established that cancerous tissues exhibit distinct spectral features, particularly in the bands associated with proteins and lipids. Therefore, molecular characteristics and possible cancer biomarkers can be easily and clearly detected via the use of a portable Raman probe spectrometer. Thus, the above-mentioned technique is able to differentiate between cancerous and normal tissue in vivo while examining a complex biological system such as that of laboratory animals. Moreover, our PCA results demonstrated effective separation between cancerous and normal tissue spectra, with specific Raman bands playing a pivotal role in this discrimination. Additionally, the 1D-CNN model with transfer learning exhibited remarkable accuracy, precision, recall, and F1 scores in identifying cancer cases, underscoring its utility in clinical applications. Altogether, this research contributes to the growing body of knowledge in cancer diagnosis and underscores the significance of spectroscopic techniques and machine learning models in advancing our understanding and detection of cancerous tissues. Consequently, the translation to clinical practice may ensure the existence of an adjuvant tool for cancer diagnosis with high specificity and sensitivity and accurate tumor margin localization in the near future.

## Figures and Tables

**Table 1:** Classification results or 1D-CNN with transfer learning model.

	Accuracy	Precision	Recall	F1-Score	Support
**control**	89.9	87.2	85	86.1	40
**cancer**		91.4	92.8	92.1	69

## Data Availability

Data are available from the corresponding author upon reasonable request.
